# Retinal and Choroidal Changes Following Implantable Collamer Lens V4c Implantation in High Myopia Patients—A 1-Year Follow-Up Study

**DOI:** 10.3390/diagnostics13193097

**Published:** 2023-09-29

**Authors:** Qiaoling Wei, Xianjin Zhou, Weiteng Chang, Rui Jiang, Xingtao Zhou, Zhiqiang Yu

**Affiliations:** 1 Department of Ophthalmology, Eye and ENT Hospital, Shanghai Medical College, Fudan University, Shanghai 200031, China; qiaoling.wei@fdeent.org (Q.W.); 20111260016@fudan.edu.cn (X.Z.); 20211260033@fudan.edu.cn (W.C.); rui.jiang@fdeent.org (R.J.); 2 Shanghai Key Laboratory of Visual Impairment and Restoration, Fudan University, Shanghai 200031, China; 3 Ocular Trauma Center, Eye and ENT Hospital, Shanghai Medical College, Fudan University, Shanghai 200031, China; 4 Department of Ophthalmology, Shanghai Children’s Medical Center, School of Medicine, Shanghai Jiao Tong University, Shanghai 200127, China

**Keywords:** implantable collamer lens, ICL, retinal thickness, choroidal thickness, choroidal vascular index, enhanced depth imaging optical coherence tomography

## Abstract

This prospective study aimed to evaluate the impact of Visian Implantable Collamer Lens (ICL) V4c implantation on retinal and choroidal morphology in patients with high myopia. A total of 97 eyes from 52 high myopic patients who underwent ICL V4c implantation were followed up for 12 months. Preoperative and postoperative evaluations included comprehensive ophthalmic assessments and enhanced depth imaging optical coherence tomography (EDI-OCT) to analyze changes in central retinal thickness (CRT), retinal volume (CRV), choroidal thickness (ChT), total choroidal area (TCA), luminal area (LA), and choroidal vascular index (CVI). Repeated measures mixed-effects models were used for comparing pre- and postoperative measurement variables and exploring relationships among age, axial length (AL), spherical equivalent refraction (SER), and postoperative retinal and choroidal changes, with statistical significance set at *p* < 0.05. Follow-up assessments were conducted at various time points, with participation rates ranging from 21% to 98%. Baseline characteristics showed a median age of 26.7 years, −10.14 diopters of SER, and an AL of 27.44 mm. Throughout the 12-month follow-up, CRT and 3.0 mm CRV consistently increased compared to the baseline, with statistically significant rises observed at postoperative day 1, week 1, and month 12. Most ChT measurements, including subfoveal ChT, declined over the 12 months, except at postoperative 6 months. Horizontal and vertical TCA and LA values significantly increased throughout the follow-up, except for month 6. After surgery, both horizontal and vertical CVI parameters exhibited an increase compared to the baseline, with some changes reaching statistical significance. Correlation analysis performed by repeated measures mixed-effects models showed that no relationship was found between age, AL, and SER and changes in postoperative retinal parameters and CVI parameters. However, postoperative changes in ChT and choroidal area parameters showed a negative correlation with AL and a positive correlation with SER. Our research demonstrated that ICL V4c implantation resulted in noteworthy alterations in retinal and choroidal morphology over a 1-year follow-up period. Moreover, in patients with high myopia, individuals with longer AL and higher degrees of myopia exhibited more pronounced postoperative changes in the choroid and retina. Further studies with extended follow-up durations are necessary to comprehensively understand the long-term effects of ICL implantation on retinal and choroidal morphology and function.

## 1. Introduction

Implantable Collamer Lens (ICL) surgery is becoming an increasingly popular choice for individuals with high myopia and low corneal thickness who are not eligible for corneal refractive surgery. Numerous studies have demonstrated the safety, effectiveness, and predictability of ICL surgery for moderate to high myopia correction, characterized by stable visual and refractive outcomes and low adverse event rates [1,2,3]. The Visian Implantable Collamer Lens (Staar Surgical AG, Nidau, Switzerland), with a central port design (V4c) and a posterior chamber phakic intraocular lens (IOL) has been extensively utilized for high myopia correction since its commercial availability in 2011. Compared to prior ICL models, the V4c model has exhibited a substantial reduction in reported complications due to enhanced aqueous humor flow and preserved anterior segment physiology [4,5,6]. However, limited research exists on the impact of the V4c model on retinal and choroidal morphology, which are vital for maintaining patients’ visual function [7,8,9,10,11,12]. 

Measurements of central retinal thickness (CRT) and choroidal thickness (ChT) aid in diagnosing and monitoring various ocular conditions. In this regard, optical coherence tomography (OCT) with enhanced depth imaging (EDI-OCT) has emerged as a valuable tool, enabling precise imaging and the quantitative assessment of CRT, central retinal volume (CRV), and ChT. Moreover, the use of image binarization technology in EDI SD-OCT images has facilitated the determination of luminal area (LA) and total choroidal area (TCA). Leveraging this information, the choroidal vascularity index (CVI) can be calculated as the LA/TCA ratio, offering a reliable OCT-based method for analyzing choroidal perfusion [13,14,15]. CVI, as a quantitative parameter, plays a significant role in understanding the vascular changes in the choroid, which is crucial for the maintenance of retinal health. Changes in CVI are associated with various ocular pathologies, including myopia and age-related macular degeneration (AMD) [16,17,18]. However, to our knowledge, few reports have explored the changes in CVI following ICL V4c implantation.

Despite the importance of these retinal and choroidal parameters, only limited reports have explored their changes following ICL V4c implantation. Thus, our study seeks to bridge this gap by comprehensively assessing the effects of ICL V4c implantation on retinal and choroidal morphology in high myopic patients. Via a prospective investigation, we intend to shed light on the potential alterations in CRT, CRV, ChT, TCA, and CVI over a 12-month follow-up period. By filling this knowledge gap, we hope our findings will contribute valuable insights to the field and provide a better understanding of the long-term consequences of ICL implantation on retinal and choroidal morphology and function.

## 2. Materials and Methods

### 2.1. Subjects

The EYE & ENT Hospital of Fudan University Institutional Review Board approved this observational study, which followed a prospective and consecutive design and adhered to the principles of the Declaration of Helsinki. All patients provided informed consent prior to their participation in the study. The study included 97 eyes from 52 high myopic patients who underwent ICL V4c implantation between September 2019 and January 2020. In this study, high myopia was defined as spherical equivalent refraction (SER) ≤ −6.00 D, which was calculated as the spherical value plus half of the cylindrical value. Patients aged between 17 and 45 years, with stable refractive error (SER ≤ −6.00 D) and no contact lens usage for at least two weeks were included in the study. The inclusion criteria also required that the endothelial cell density (ECD) was greater than 2000 cells/mm^2^. Exclusion criteria comprised of ocular diseases such as corneal dystrophy, cataract, chorioretinal disease, glaucoma or optic nerve diseases, a history of ocular inflammation or trauma, a history of systemic diseases, or previous ocular surgeries.

### 2.2. Preoperative Examinations

All patients underwent routine preoperative examinations, including best-corrected visual acuity (BCVA), SER, slit-lamp examination, fundus examination after pupillary dilation, non-contact intraocular pressure (IOP) measurement (Canon Full Auto Tonometer TX-F; Canon, Inc., Tokyo, Japan), axial length (AL), and ECD (SP-2000P; Topcon Corporation, Kyoto, Japan). Using a Pentacam HR (Oculus Optikgerate Wetzlar, Wetzlar, Germany), corneal thickness, corneal diameter (white-to-white), and anterior chamber parameters were measured. A Spectralis HRA-OCT (Heidelberg Engineering, Heidelberg, Germany) was used to obtain horizontal and vertical EDI-OCT scans that were centered on the fovea of each eye.

### 2.3. Surgical Technique

The surgical procedures were skillfully performed by Dr. Y.Z.Q. at the Department of Ophthalmology, EYE & ENT Hospital of Fudan University, following established protocols for the surgical technique, intraocular lens (IOL) design, and power calculation. Prior to the surgery, patients were administered ophthalmic antibiotic instillation for 3 days (four times a day) and received dilating drops and topical anesthesia on the day of the procedure. The anterior chamber was infused with 0.2 mL of sodium hyaluronate (15 mg/mL mass concentration, supplied by Shanghai Qisheng Biological Agents Co., Ltd., Shanghai, China), which was entirely aspirated at the conclusion of the surgery. Subsequently, an ICL V4c was inserted through a 3 mm clear corneal incision using an injector cartridge. The ICL V4c haptics were securely fixed into the ciliary sulcus using a manipulator, and any residual viscoelastics were thoroughly washed out of the anterior chamber with a balanced salt solution. Following the surgery, patients were prescribed antibiotics and steroid eye drops to be administered four times a day for 2 weeks, with a gradual tapering of the dosage thereafter [19].

### 2.4. Postoperative Follow-Up

**Figure 1:**
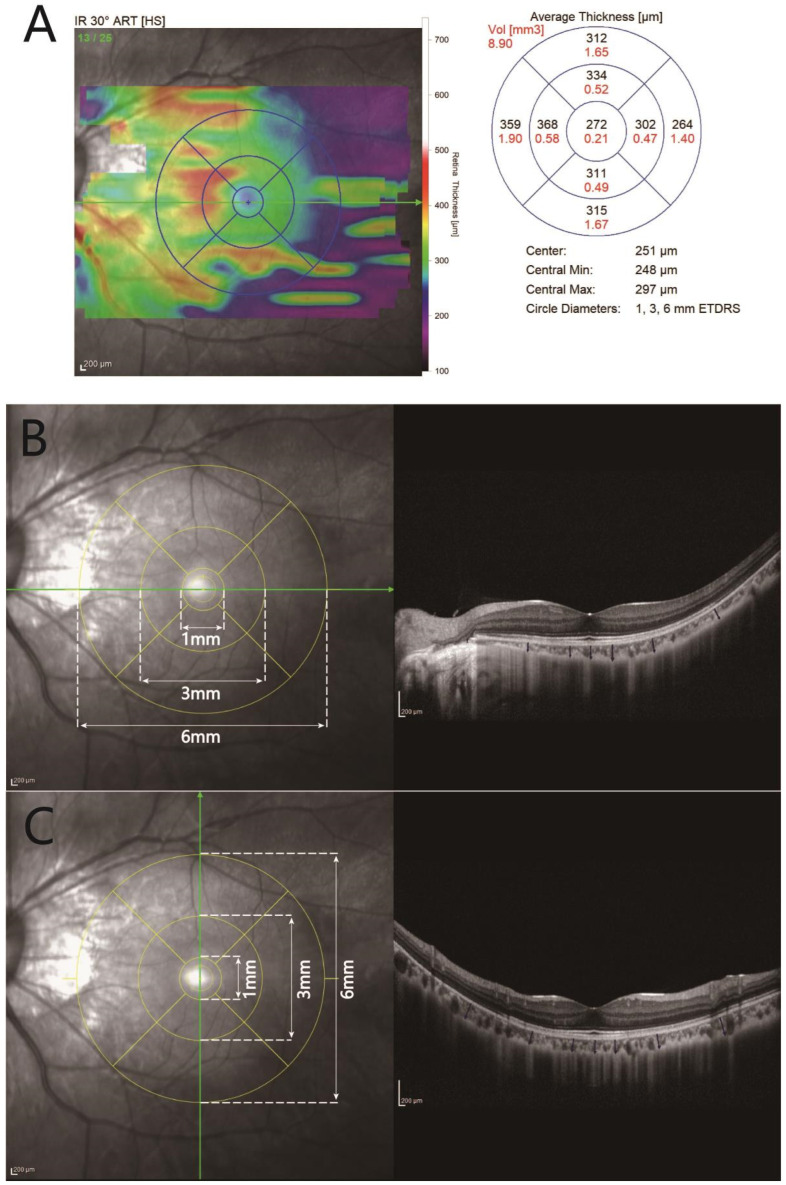
Retinal and choroidal thickness measurements. (**A**): CRT and central retinal volumes (1 mm CRV, 6 mm CRV) were obtained using the built-in software, utilizing an ETDRS grid based on a retinal thickness map. (**B**,**C**) Choroidal thickness was assessed by creating an ETDRS-style topographic map with the inbuilt software of Heidelberg Spectralis (version 2.5.5, Copyright 2021). Manual measurements were performed at specific locations in the EDI-OCT image, including the subfoveal area and distances of 0.5 mm, 1.5 mm, and 3.0 mm from the fovea in the temporal, nasal, superior, and inferior directions, using provided calipers.

Postoperative follow-up visits were scheduled at 1 day, 1 week, and 1, 3, 6, and 12 months. The follow-up visits included assessments of the following: (1) BCVA, SER, IOP; (2) CRT, CRV, ChT, TCA, and CVI parameters from raw EDI-OCT images. In the postoperative period, OCT images were obtained using the “follow-up” function to ensure consistent scanning of the same choroidal areas. CRT and CRV values within 3 mm (3 mm CRV) and 6 mm (6 mm CRV) diameters centered over the fovea were calculated using built-in software. ChT was measured as the distance between the retinal pigment epithelium–Bruch’s membrane complex and the choroid–scleral junction using a manual caliper function of the built-in software. Upon generating an ETDRS-style topographic map using built-in software, ChT at 0.5 mm, 1.5 mm, and 3.0 mm from the fovea in temporal, nasal, superior, and inferior locations was also measured (detailed in Figure 1). For the analysis of choroidal vascularity, we selected the subfoveal area within 3 mm and 6 mm diameters, centered over the fovea (1.5 mm or 3 mm on the side of the fovea) from the raw OCT images converted into 8-bit pixel resolution. Using Niblack autolocal thresholding, we obtained the mean pixel value for all points and applied the color threshold tool to distinguish the LA and the stromal area (SA) within the selected 3 mm and 6 mm areas. The TCA, LA, and SA values were recorded on both horizontal and vertical scans. The CVI was calculated as the ratio of LA to TCA. All OCT images were interpreted by two experienced technicians three times. In cases of disagreement, a third technician was consulted to interpret the results.

### 2.5. Statistical Analysis

The Statistical Package for the Social Sciences (version 23.0, SPSS, Inc., Chicago, IL, USA) was used for statistical analysis, with data presented as mean ± standard deviation. To compare pre- and postoperative measurement variables, a repeated measures mixed-effects model was employed with statistical significance defined as *p* < 0.05 [20]. Repeated measures mixed-effects model was also used to evaluate the relationships between age, AL, SER, and changes in retinal and choroidal parameters after surgery.

## 3. Results

In this prospective study, 97 eyes from 52 patients were included, with an average age at diagnosis of 26.7 ± 6.2 years (range, 17–44 years), in which 41 of the patients were female. The clinical and demographic characteristics of patients prior to surgery are presented in Table 1. The average number of postoperative follow-up visits was 3.01 ± 1.28 (range, 1–6). Follow-up visits occurred at 1 day, 1 week, and at 1, 3, 6, and 12 months after surgery, with 51, 36, 27, 26, 11, and 15 patients returning for each respective time point. All patients exhibited postoperative intraocular pressure below 21 mmHg, and their uncorrected distance visual acuity (UDVA) either met or surpassed the baseline BCVA levels at each follow-up. No intraoperative complications were encountered, and no eyes necessitated ICL removal or exchange during the study. No vision-threatening complications, such as anterior subcapsular opacity, cataract, secondary glaucoma, pupillary block, or others that could have influenced the study outcomes were reported during the entire follow-up period.

**Figure 2:**
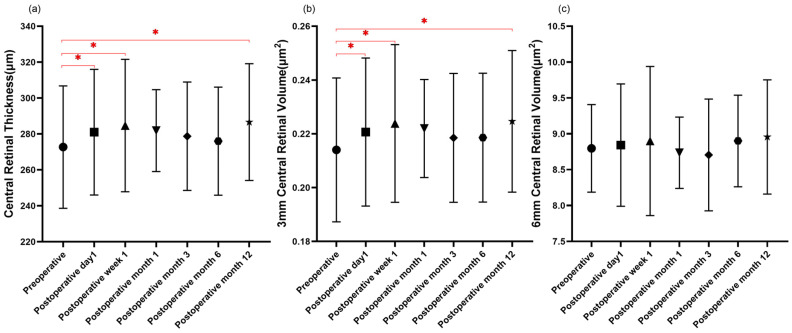
Postoperative changes in central retinal thickness and volume. (**a**,**b**): The measurements of central retinal thickness and central retinal volume with 3.0 mm central at the fovea at postoperative day 1 (*n* = 51), week 1 (*n* = 36), and month 12 (*n* = 15) were significantly thicker than baseline values (* *p* < 0.05). (**c**): No significant changes were found in central retinal volume with 6.0 mm central at the fovea after the surgery. Data presented as mean ± standard deviation. The symbols, including Circles, Squares, Triangles, Inverted Triangles, Diamonds, Hexagons, and Stars, correspond to data points at specific time intervals along the X-axis of graph. Statistical significance was determined using a repeated measures mixed-effects model. * *p* < 0.05 compared to baseline or other time points as indicated.

Throughout the entire follow-up period, postoperative measurements of CRT and 3.0 mm CRV consistently exhibited a rise compared to the baseline values. Notably, statistically significant increases were observed at postoperative day 1, week 1, and month 12. In contrast, 6 mm CRV values at postoperative day 1, week 1, month 6, and month 12 were slightly thicker than preoperative values but were not statistically significant, while 6 mm CRV measurements at postoperative month 1 and month 3 were thinner than the preoperative baseline values and also lacked statistical significance (detailed in Table 2 and Figure 2).

**Figure 3:**
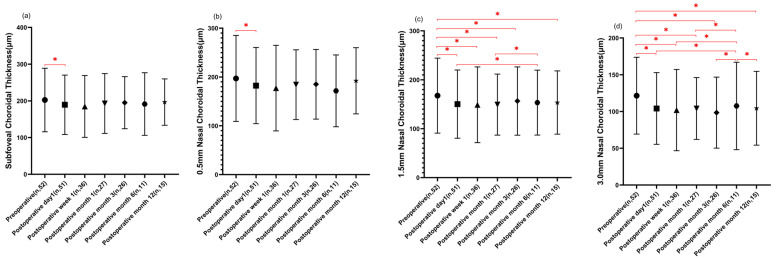

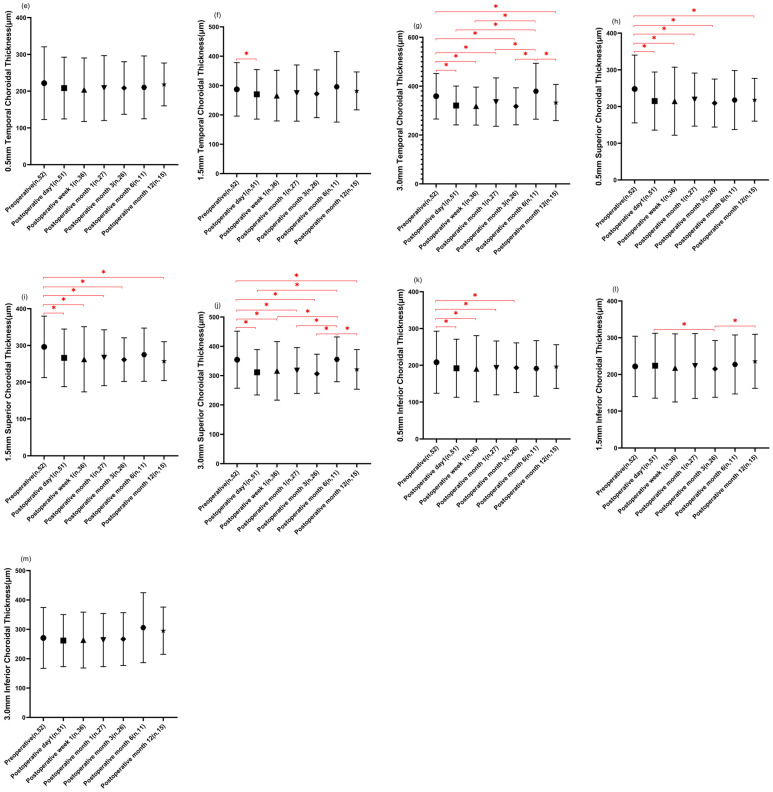
Postoperative changes in choroidal thickness at different time points. (**a**,**b**,**f**): The measurements of subfoveal 0.5 mm nasal and 1.5 temporal choroidal thickness at postoperative day 1 (*n* = 51) were significantly thinner than baseline (*n* = 52) (* *p* < 0.05). (**c**): The measures of all postoperative 1.5 mm nasal choroidal thickness were thinner than baseline with statistical significance, except for postoperative month 6 (*n* = 11). The measures of postoperative month 6 (*n* = 11) were thicker than postoperative day 1 (*n* = 51) and month 1 (*n* = 27) with statistical significance (* *p* < 0.05). (**d**,**g**,**j**): The 3.0 mm nasal, temporal, and superior choroidal thickness measurements of preoperative and postoperative month 6 (*n* = 11) were thicker than measurements of other operative points with statistical significance (* *p* < 0.05). (**e**,**m**): No significant changes were found in 0.5 mm temporal and 3.0 mm choroidal thickness after the surgery. (**h**,**i**): The 0.5 mm and 1.5 mm superior choroidal thickness measurements of preoperative were thicker than measurements of other operative points with statistical significance, except for the postoperative month 6 (*n* = 11) (* *p* < 0.05). (**l**): The measurements of 1.5 mm inferior choroidal thickness at postoperative month 3 (*n* = 26) were significantly thinner than those at postoperative day 1 (*n* = 51) and month 12 (*n* = 15) (* *p* < 0.05). (**k**): The 0.5 mm inferior choroidal thickness significant reductions were evident at postoperative day 1 (*n* = 51), week 1 (*n* = 36), month 1 (*n* = 27), and month 3 (*n* = 26) compared to the baseline values (* *p* < 0.05). Data presented as mean ± standard deviation. The symbols, including Circles, Squares, Triangles, Inverted Triangles, Diamonds, Hexagons, and Stars, correspond to data points at specific time intervals along the X-axis of graph. Statistical significance was determined using a repeated measures mixed-effects model. * *p* < 0.05 compared to baseline or other time points as indicated.

Regarding the ChT parameters, subfoveal ChT initially decreased from the baseline (202.8 ± 86.5 µm) to day 1 (189.4 ± 80.9 µm) and week 1 (184.9 ± 84.2 µm). Subsequently, there was a gradual increase (192.9 ± 81.5 µm at month 1, 196.7 ± 63.1 µm at month 12), although it did not return to the baseline values. Notably, a statistically significant reduction was observed on day 1 compared to the baseline. Similar patterns were observed for the 0.5 mm nasal ChT, which initially decreased at day 1 (182.4 ± 78.0 μm) and week 1 (177.2 ± 87.7 μm), followed by a gradual increase thereafter (192.3 ± 67.8 μm at month 12). However, it did not return to the baseline values, and a statistically significant reduction was observed on day 1 compared to the baseline. As for the 1.5 mm and 3.0 mm nasal ChT, they also displayed an initial significant decrease at day 1 (150.3 ± 69.8 μm vs. 104.2 ± 48.8 μm) and week 1 (148.9 ± 77.4 μm vs. 102.0 ± 55.3 μm), followed by a slight increase at month 12 (153.6 ± 64.7 μm vs. 104.4 ± 50.2 μm), although they remained thinner than the baseline values (167.7 ± 76.6 μm vs. 121.5 ± 52.2 μm). Notably, statistically significant reductions were observed at all postoperative time points when compared to the baseline measurements, with the exception of month 6. However, the 0.5 mm, 1.5 mm, and 3.0 mm temporal ChT initially exhibited a slight decrease at day 1 (208.5 ± 83.9 μm vs. 270.2 ± 84.3 μm vs. 321.0 ± 79.5 μm) and week 1 (204.0 ± 86.4 μm vs. 265.7 ± 86.3 μm vs. 318.5 ± 77.9 μm), followed by a slight increase thereafter but did not return to the baseline values (221.9 ± 98.8 μm vs. 287.2 ± 91.0 μm vs. 358.9 ± 93.3 μm) when assessed at month 12 (218.4 ± 58.3 μm vs. 281.8 ± 64.6 μm vs. 333.1 ± 74.1 μm). Notably, statistically significant reductions were observed at postoperative day 1 compared to the baseline values in the 1.5 mm temporal ChT. However, statistically significant reductions at all postoperative time points compared to the baseline, except for month 6, were observed in the 3.0 mm temporal ChT. Regarding the superior ChT (0.5 mm, 1.5 mm, and 3.0 mm), consistent decreases were observed throughout the follow-up period with statistical significance, except for month 6. As for the 0.5 mm inferior ChT, significant reductions were evident at postoperative day 1 (192.0 ± 79.0 μm), week 1 (190.7 ± 90.1 μm), month 1 (192.8 ± 73.3 μm), and month 3 (193.4 ± 67.6 μm) compared to the baseline values (208.3 ± 84.4 μm). Notably, the 1.5 mm inferior ChT measurements at postoperative month 3 (215.1 ± 77.4 μm) were significantly thinner than those at postoperative day 1 (223.8 ± 88.4 μm) and month 12 (235.8 ± 73.6 μm). No statistical significance was observed for the 3.0 mm inferior ChT before and after surgery (detailed in Table 3 and Figure 3).

In this study, we assessed the TCA, LA, and SA within 3 mm and 6 mm diameters centered over the fovea, both horizontally and vertically. The results showed a significant increase in 3.0 mm horizontal and vertical TCA and LA, as well as 6.0 mm horizontal and vertical TCA, LA, and SA during the follow-up period compared to their respective preoperative baselines, except for month 6. The peak values were observed at postoperative day 1, followed by a subsequent decrease over time. Notably, the 3.0 mm horizontal SA exhibited significant increases at day 1, week 1, month 1, and month 3 compared to the baseline values, with postoperative day 1 measurements being larger than those at month 6. Similarly, the 3.0 mm vertical SA showed significant increases at postoperative day 1, day 7, and month 1, followed by a subsequent decrease, ultimately returning to baseline levels at postoperative month 12 (detailed in Table 4).

**Figure 4:**
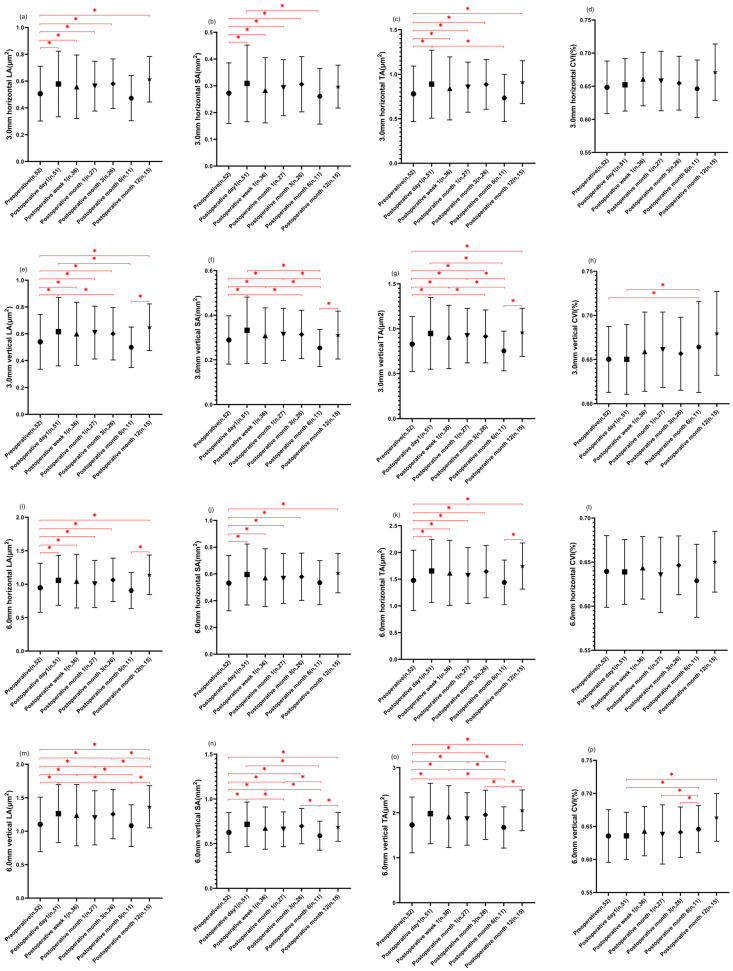
Changes in choroidal area and choroidal vascular index parameters at different time points. (**a**,**j**): The preoperative (*n* = 52) measurements of 3.0 mm horizontal LA and 6.0 mm horizontal SA were significantly smaller than the measures of all postoperative points, except for postoperative month 6 (*n* = 11) (* *p* < 0.05). (**b**): The preoperative (*n* = 52) measurements of 3.0 mm horizontal SA were significantly smaller than the measurements of postoperative day 1 (*n* = 51), week 1 (*n* = 36), month 1 (*n* = 27), and month 3 (*n* = 26). The measures of postoperative day 1 (*n* = 51) were significantly larger than postoperative month 6 (*n* = 11) (* *p* < 0.05). (**c**): The preoperative (*n* = 52) measurements of 3.0 mm TA were significantly smaller than the measurements of all postoperative points, except for postoperative month 6 (*n* = 11). The measurements of postoperative day 1 (*n* = 51) were significantly larger than postoperative month 6 (*n* = 11) (* *p* < 0.05). (**d**,**l**): No significant changes were found in 3.0 mm and 6.0 mm horizontal CVI after the surgery. (**e**): The preoperative (*n* = 52) measurements of 3.0 mm vertical LA were significantly smaller than the measurements of all postoperative points, except for postoperative month 6 (*n* = 11). The measurements of postoperative day 1 (*n* = 51) were significantly larger than postoperative month 3 (*n* = 26) and 6 (*n* = 11), and the measurements of postoperative month 12 (*n* = 15) were significantly larger than postoperative month 6 (*n* = 11) (* *p* < 0.05). (**f**): The preoperative (*n* = 52) measurements of 3.0 mm vertical SA were significantly smaller than the measurements of postoperative day 1 (*n* = 51), week 1 (*n* = 36), and month 1 (*n* = 27). The measurements of postoperative day 1 (*n* = 51) were significantly larger than postoperative month 3 (*n* = 26) and month 6 (*n* = 11), and the measurements of postoperative month 6 (*n* = 11) were significantly smaller than the measurements of postoperative week 1 (*n* = 36), month 1 (*n* = 27), and 12 (*n* = 15) (* *p* < 0.05). (**g**): The preoperative (*n* = 52) measurements of 3.0 mm vertical TA were significantly smaller than the measurements of all postoperative points, except for month 6 (*n* = 11). The measurements of postoperative day 1 (*n* = 51) were significantly larger than postoperative month 3 (*n* = 26) and month 6 (*n* = 11), and the measurements of postoperative month 6 (*n* = 11) were significantly smaller than the measurements of postoperative week 1 (*n* = 36), month 1 (*n* = 27), and 12 (*n* = 15) (* *p* < 0.05). (**h**): The 3.0 mm vertical CVI measures of postoperative month 6 (*n* = 11) were significantly higher than the measures of preoperative (*n* = 52) and postoperative day 1 (*n* = 51) (* *p* < 0.05). (**i**) and (**k**): The preoperative (*n* = 52) measures of 6.0 mm horizontal LA and TA were significantly smaller than the measures of all postoperative points, except for month 6. The measurements of postoperative month 6 (*n* = 11) were significantly smaller than postoperative month 12 (*n* = 15) (* *p* < 0.05). (**m**): The preoperative (*n* = 52) measurements of 6.0 mm vertical LA were significantly smaller than the measures of all postoperative points, except for month 6 (*n* = 11). The measurements of postoperative month 6 (*n* = 11) were significantly smaller than postoperative day 1 (*n* = 51), week 1 (*n* = 36), and month 12 (*n* = 15), and the measurements of month 12 (*n* = 15)were significantly larger than postoperative month 1 (*n* =27) and month 3 (*n* = 26) (* *p* < 0.05).(**n**): The preoperative (*n* = 52) measurements of 6.0 mm vertical SA were significantly smaller than the measures of all postoperative points, except for month 6 (*n* = 11). The measurements of postoperative day 1 (*n* = 51) were significantly larger than the measures of month 1 (*n* = 27), and month 6 (*n* = 11), and the measurements of month 1 (*n* = 27) were significantly larger than postoperative month 6 (*n* = 11). The measurements of month 6 (*n* = 11) were significantly smaller than postoperative month 6 (*n* = 26), and month 12 (*n* = 15) (* *p* < 0.05). (**n**): The preoperative (*n* = 52) and postoperative month 6 measurements of 6.0 mm vertical TA were significantly smaller than the measures of all postoperative points (* *p* < 0.05). (**o**): The preoperative (*n* = 52) and postoperative month 6 (*n* = 11) measurements of 6.0 mm vertical TA were significantly smaller than the measures of all postoperative points. (* *p* < 0.05). (**p**): The 6.0 mm vertical CVI measures of postoperative month 6 were significantly higher than the measures of postoperative day 1, month 1, and month 3. The measurements of postoperative month 12 were significantly higher than postoperative day 1 (* *p* < 0.05). (**p**): The 6.0 mm vertical CVI measures of postoperative month 6 (*n* = 11) were significantly higher than the measures of postoperative day 1 (*n* = 51), month 1 (*n* = 27), and month 3 (*n* = 26). The measurements of postoperative month 12 (*n* = 15) were significantly higher than postoperative day 1 (* *p* < 0.05). The symbols, including Circles, Squares, Triangles, Inverted Triangles, Diamonds, Hexagons, and Stars, correspond to data points at specific time intervals along the X-axis of graph. Data presented as mean ± standard deviation. Statistical significance was determined using a repeated measures mixed-effects model. * *p* < 0.05 compared to baseline or other time points as indicated. CVI, choroidal vascular index; TCA, total choroidal area; LA, luminal area; SA, stromal area.

A marginal rise in the 3.0 mm horizontal CVI was observed post-surgery, except for the 6-month mark where the difference lacked statistical significance. Conversely, the 3.0 mm vertical CVI displayed a significant increase at postoperative month 6 compared to the baseline and postoperative day 1. However, no significant differences were found among the 6.0 mm horizontal CVI parameters before and after surgery. Similarly, the 6.0 mm vertical CVI exhibited a significant increase post-surgery, with peak values observed at postoperative month 6 and month 12 (detailed in Table 4 and Figure 4).

To provide a more precise assessment of the surgical impact, we concentrated on two pivotal time points: the baseline to post-week 1 and post-month 1 to post-month 12. In the comparison between the baseline and post-week 1, which captures short-term effects, we analyzed data from 69 eyes of 35 patients who completed both the preoperative and post-week 1 assessments using a repeated measures mixed-effects model. Our analysis revealed an increase in retinal thickness and volume, accompanied by a decrease in all choroidal thickness parameters. For the comparison between post-month 1 and post-month 12, elucidating medium- to long-term effects, we employed data from 19 eyes of 10 patients who completed both the post-month 1 and post-month 12 assessments via the same analysis method. In this context, we observed an increase in both retinal and choroidal thickness and volume, signifying an overall expansion of these parameters over the medium to long term (detailed in Table 5).

We employed a repeated measures mixed-effects model to assess the correlation between age, AL, and SER with changes in retinal and choroidal parameters post-surgery using a significance level of *p* < 0.05. Our analysis revealed that postoperative changes in all TCA, SA, and LA parameters were negatively correlated with AL, except for the 3.0 mm vertical SA, which exhibited a positive correlation with SER. Moreover, subfoveal ChT, 0.5 mm and 3.0 mm temporal, 0.5 mm superior, 0.5 mm and 1.5 mm nasal, and 0.5 mm inferior ChT showed negative correlations with AL. On the other hand, postoperative changes in subfoveal ChT, all superior ChT, 1.5 mm and 0.5 mm temporal ChT, and 3.0 mm nasal ChT parameters were positively related to SER. However, we found no significant associations between any of the CVI parameters, retinal parameters (CRT, 3.0 mm and 6.0 mm CRV), 1.5 mm and 3.0 mm inferior ChT with age, SER, and AL. Additionally, age did not exhibit significant correlations with any of the postoperative changes in retinal and choroidal parameters.

## 4. Discussion

The implantation of ICL V4c is shown to be a safe and effective surgical option for correcting myopia, as evidenced by its stable and exceptional visual and refractive outcomes, as well as a low incidence of adverse events [19,21,22]. To our knowledge, there is limited research on the impact of the V4c model on the retina and choroid, which play crucial roles in maintaining patients’ visual acuity. Furthermore, retinal and choroidal blood vessels in patients with high myopia may exhibit increased susceptibility to intraocular surgery as compared to those without high myopia [23]. Monitoring these changes can offer valuable information about postoperative outcomes and help clinicians identify patients who may need additional intervention or follow-up care. Consequently, we conducted a prospective study to investigate changes in retinal and choroidal parameters following ICL V4c implantation in patients with high myopia.

In our study, we observed a consistent increase in retinal thickness and volume following ICL V4c implantation throughout the entire follow-up period, with notable changes noted on day 1, day 7, and month 12 post-surgery. Interestingly, these changes were not found to be associated with SER, AL, or age. Our findings align with those of Pachon et al., who reported increased macular thickness at postoperative months 1 and 12 following ICL V4c implantation [7]. Conversely, Xu et al. found no significant changes in central retinal thickness (CRT) at postoperative weeks 1, 3, and 6, as well as blood flow density of superficial and deep macular fovea [24]. The underlying causes of CRT increase following ICL implantation remain a subject of uncertainty. Postoperative increases in macular thickness following femtosecond LASIK were attributed to potential pathogenesis such as corneal shape alteration, subclinical macular edema, magnification changes, optical artifacts, errors in OCT analysis, or variations of intraocular pressure (IOP) [25,26,27]. In the case of monofocal and trifocal intraocular lens (IOL) implantation for cataract surgery, postoperative increases in macular thickness were proposed to be due to inflammation, vitreous volume changes, or variations in repeatability and reproducibility [28,29,30,31]. Our comprehensive analysis revealed that the mean value of preoperative and postoperative differences in CRT ranged from 0.38 to 12.18 µm, accounting for less than 5% of total retinal thickness. Combining the results of baseline vs. post-week 1 and post-month 1 vs. post-month 12, we suggest that ICL implantation may induce subclinical macular changes that persist up to 12 months after surgery. However, it is crucial to note that these observed differences, although statistically significant at specific time points, may not have significant clinical implications. Further investigations with longer follow-up durations and a more extensive battery of macular function tests are needed to determine the duration and functional significance of these observed changes.

The choroid is a vital part of the eye, and changes in its thickness and vascularity can have a significant impact on refractive power and visual quality, especially in patients with high myopia [18]. The increase in choroidal thickness during myopic recovery in chicks and pediatric patients wearing overnight orthokeratology contact lenses demonstrates the choroid’s compensatory role in these conditions [32,33]. ICL surgery is another treatment for myopia, and studies have shown varying effects on choroidal thickness. As such, it is crucial to investigate these changes to optimize visual outcomes and long-term ocular health for patients undergoing ICL surgery.

Recent research has explored the impact of various refractive surgeries on ChT. Most of these studies typically involve a 3-month postoperative observation period, during which ChT was reported to increase at 2 h and 3 months after surgery [10,11,12]. However, our study reveals a departure from these established findings. Contrary to previous research, we observed a decline in ChTs, including subfoveal ChT, particularly evident on postoperative day 1. These observations were substantiated via a detailed analysis of baseline vs. post-week 1 changes (detailed in Table 5). Notably, ChT in the superior quadrant displayed heightened susceptibility to surgical influence, indicated by significant thinning at multiple postoperative time points (excluding 6 months postoperatively). Furthermore, our correlation analysis unveiled that postoperative ChT changes exhibit a negative association with patients’ AL and a positive correlation with SER. These results underscore the potential influence of patient demographics on postoperative ChT changes, possibly contributing to the disparities between our findings and those of other researchers. Additionally, variations in imaging equipment could also play a role. While prior studies employed swept-source optical coherence tomography (SS-OCT) from Topcon, our research employed Spectralis HRA-OCT from Heidelberg Engineering. The question of whether ICL implantation leads to image variations remains an open one. Examining the results of post-month 1 vs. post-month 12, we observed an increase in ChT from post-month 1 to post-month 12. Collectively, our findings suggest that patients may undergo choroidal remodeling following ICL surgery. This highlights the need for diligent consideration in clinical practice.

The values of ChT can be influenced by various factors, including age, sex, AL, and SER. Furthermore, ChT measurements may exhibit regional variability and susceptibility to segmentation errors, leading to limited reproducibility [34]. In contrast, the CVI remains unaffected by age, sex, SER, AL, and other biometric parameters, rendering it a more robust metric than choroidal thickness values [18,34,35]. In our study, we observed a significant increase in postoperative choroidal area compared to the baseline, with the peak augmentation evident 1 day after surgery. CVI also displayed a notable increase, although not all of these changes reached statistical significance, implying potential alterations in choroidal perfusion and vascularization following the surgical procedure. The postoperative changes in all 3.0 mm and 6.0 mm TCA, SA, and LA parameters were found to be negatively correlated to AL, except for 3.0 mm SA, which exhibited a positive correlation with SER. Meanwhile, no significant connections were observed between any of the CVI parameters and age, SER, and AL. Thus, our findings emphasize the importance of evaluating not only ChT but also choroidal area and CVI to comprehensively assess postoperative choroidal changes. However, the clinical significance of these changes still needs to be confirmed by future studies with larger cohorts of diverse populations, longer follow-up periods, and more frequent visual acuity examinations. Furthermore, exploring the association between these postoperative choroidal changes and clinical outcomes, such as postoperative refractive stability and visual acuity, could further enhance our knowledge and guide clinical decision-making in the management of high myopia.

Our study’s advantage lies in our comprehensive measurement of multiple retinal and choroidal parameters, including CRT, CRV, ChT, TCA, and CVI, via the use of EDI-OCT technology. This extensive assessment allowed us to obtain valuable insights into the dynamics of these parameters over a 12-month follow-up period. To our knowledge, our study is among the few that have simultaneously investigated the impact of ICL V4c on both the retina and choroid.

Nevertheless, our study does come with several limitations. Firstly, our sample size was relatively small, and we encountered some instances of patients not returning for follow-up at the 6 and 12-month marks. These factors may have influenced the statistical power of our study. Secondly, it is important to note that our study exclusively enrolled patients with high myopia, limiting the generalizability of our results to individuals with other refractive errors. Future research endeavors should consider larger and more diverse patient populations, coupled with extended follow-up durations, to gain deeper insights into the long-term alterations in retinal and choroidal parameters post ICL V4c implantation and to elucidate the underlying mechanisms driving these changes. Furthermore, we acknowledge another limitation within our study: all surgical procedures were conducted by the same surgeon. Unfortunately, we did not record the duration of each surgery, hindering our ability to explore the potential influence of surgical maneuvers on retinal and choroidal parameters. ICL V4c implantation surgery is classified as intraocular surgery, and while the entire procedure, including viscoelastic injection and aspiration, typically lasts less than 15 min, it may still disrupt intraocular fluid dynamics. Consequently, this disruption could exert an influence on the retina and choroid to some extent. Therefore, we recommend further investigations that explicitly focus on how surgical maneuvers during ICL V4c implantation surgery may impact retinal and choroidal parameters.

## 5. Conclusions

In conclusion, our study provides important insights into the effects of ICL V4c on the retina and choroid and may help improve surgical outcomes for patients with high myopia. The findings suggest that changes in retinal and choroidal values may be most pronounced in the first 6 months after surgery, and postoperative months 3, 6, and 12 may be critical time points for monitoring changes in these parameters. Furthermore, in patients with high myopia, individuals with longer AL and higher degrees of myopia exhibited more pronounced postoperative changes in the choroid and retina. Further research in this area can provide valuable insights into the underlying mechanisms driving these changes and can help clinicians optimize postoperative outcomes for their patients.

## Figures and Tables

**Table 1:** Summary of clinical and demographic features—preoperative measurements.

Parameter	Total or Mean (Range)
No. of patients	52
No. of eyes (Right/Left)	97 (49/48)
Gender (*n*, male/female)	11/41
Age (yrs)	26.7 ± 6.2 (17~44)
Spherical equivalent refraction (D)	−10.14 ± 2.8 (−18.25~−6)
Best-corrected visual acuity (logMAR)	0.03 ± 0.07 (−0.08~0.40)
Axial length (mm)	27.44 ± 1.3 (24.76~30.75)
Central corneal thickness (μm)	514.47 ± 30.65 (438~630)
Anterior chamber depth (mm)	3.28 ± 0.32 (2.69~4.03)
Lens thickness (mm)	3.72 ± 0.27 (3.21~4.64)
Corneal endothelial cell density (cell/mm^2^)	2651.96 ± 245.42 (2144~3287)
Anterior chamber volume (mm^3^)	205.26 ± 31.01 (136~279)

**Table 2:** Retinal morphology parameters before and after surgery.

Parameter	Preoperative	Postoperative					
		Day 1	Week 1	Month 1	Month 3	Month 6	Month 12
Eyes (*n*)	97	93	70	47	46	21	28
CRT (μm)	272.7 ± 34.1	281.0 ± 35.0	284.7 ± 36.9	281.9 ± 22.8	278.7 ± 30.2	276.0 ± 30.1	286.6 ± 32.5
3 mm CRV (μm^2^)	0.21 ± 0.03	0.22 ± 0.03	0.22 ± 0.03	0.22 ± 0.02	0.22 ± 0.02	0.22 ± 0.02	0.22 ± 0.03
6 mm CRV (μm^2^)	8.80 ± 0.61	8.84 ± 0.85	8.90 ± 1.04	8.74 ± 0.50	8.71 ± 0.78	8.90 ± 0.64	8.96 ± 0.80

CRT, central retinal thickness; 3 mm CRV or 6 mm CRV, central retinal volume within 3 mm or 6 mm diameters centered over the fovea.

**Table 3:** Choroid thickness parameters before and after surgery.

Choroid Thickness (μm)	Preoperative	Postoperative					
		Day 1	Week 1	Month 1	Month 3	Month 6	Month 12
Eyes (*n*)	97	93	70	47	46	21	28
Subfoveal ChT	202.8 ± 86.5	189.4 ± 80.9	184.9 ± 84.2	192.9 ± 81.5	195.1 ± 71.0	191.4 ± 85.3	196.7 ± 63.1
0.5 mm nasal ChT	197.0 ± 88.11	182.4 ± 78.0	177.2 ± 87.7	184.3 ± 71.4	185.1 ± 71.4	171.6 ± 73.5	192.3 ± 67.8
1.5 mm nasal ChT	167.7 ± 76.6	150.3 ± 69.8	148.9 ± 77.4	149.3 ± 62.4	156.6 ± 69.8	153.4 ± 66.4	153.6 ± 64.7
3.0 mm nasal ChT	121.5 ± 52.2	104.2 ± 48.8	102.0 ± 55.3	104.1 ± 42.1	98.5 ± 48.3	107.6 ± 59.4	104.4 ± 50.2
0.5 mm temporal ChT	221.9 ± 98.8	208.5 ± 83.9	204.0 ± 86.4	208.4 ± 88.3	208.7 ± 71.5	210.3 ± 85.4	218.4 ± 58.3
1.5 mm temporal ChT	287.2 ± 91.0	270.2 ± 84.3	265.7 ± 86.3	274.6 ± 95.7	272.2 ± 81.4	295.8 ± 120.1	281.8 ± 64.6
3.0 mm temporal ChT	358.9 ± 93.3	321.0 ± 79.5	318.5 ± 77.9	334.9 ± 99.0	317.6 ± 75.8	379.4 ± 114.4	333.1 ± 74.1
0.5 mm Superior ChT	247.9 ± 92.4	215.0 ± 79.2	214.6 ± 92.7	219.0 ± 72.2	209.5 ± 65.4	217.8 ± 80.3	218.4 ± 58.2
1.5 mm Superior ChT	296.2 ± 83.8	266.3 ± 78.5	262.5 ± 88.8	266.7 ± 76.0	261.4 ± 59.4	275.0 ± 72.4	257.3 ± 53.0
3.0 mm Superior ChT	354.5 ± 97.4	311.5 ± 77.5	316.4 ± 100.0	317.4 ± 78.4	306.6 ± 66.7	355.6 ± 76.4	321.4 ± 67.7
0.5 mm inferior ChT	208.3 ± 84.4	192.0 ± 79.0	190.7 ± 90.1	192.8 ± 73.3	193.4 ± 67.6	191.4 ± 75.6	196.5 ± 59.5
1.5 mm inferior ChT	221.9 ± 82.2	223.8 ± 88.4	217.7 ± 92.8	222.9 ± 88.4	215.1 ± 77.4	227.1 ± 80.3	235.8 ± 73.6
3.0 mm inferior ChT	270.8 ± 103.5	261.7 ± 88.7	263.6 ± 95.2	263.5 ± 90.4	267.0 ± 89.8	305.8 ± 119.0	295.4 ± 80.5

ChT, choroidal thickness.

**Table 4:** Choroid volume before and after surgery.

Choroid Volume (mm^2^)	Preoperative	Postoperative					
		Day 1	Week 1	Month 1	Month 3	Month 6	Month 12
Eyes (*n*)	97	93	70	47	46	21	28
3.0 mm horizontal CVI	64.83 ± 4.0%	65.21 ± 4.0%	66.09 ± 4.0%	65.79 ± 4.5%	65.46 ± 4.1%	64.62 ± 4.3%	67.13 ± 4.3%
3.0 mm horizontal LA	0.506 ± 0.20	0.579 ± 0.24	0.558 ± 0.24	0.562 ± 0.19	0.580 ± 0.19	0.473 ± 0.17	0.614 ± 0.17
3.0 mm horizontal SA	0.274 ± 0.11	0.310 ± 0.14	0.283 ± 0.12	0.293 ± 0.10	0.306 ± 0.10	0.261 ± 0.10	0.297 ± 0.08
3.0 mm horizontal TCA	0.780 ± 0.31	0.889 ± 0.38	0.841 ± 0.35	0.855 ± 0.28	0.886 ± 0.28	0.734 ± 0.27	0.911 ± 0.24
3.0 mm vertical CVI	65.03 ± 3.8%	65.02 ± 3.9%	65.90 ± 4.5%	66.11 ± 4.3%	65.66 ± 4.1%	66.42 ± 5.1%	67.96 ± 4.8%
3.0 mm vertical LA	0.540 ± 0.20	0.616 ± 0.26	0.600 ± 0.24	0.609 ± 0.20	0.601 ± 0.20	0.500 ± 0.15	0.649 ± 0.17
3.0 mm vertical SA	0.289 ± 0.11	0.333 ± 0.15	0.308 ± 0.12	0.314 ± 0.12	0.314 ± 0.11	0.253 ± 0.08	0.311 ± 0.11
3.0 mm vertical TCA	0.829 ± 0.31	0.949 ± 0.40	0.908 ± 0.35	0.923 ± 0.30	0.915 ± 0.29	0.753 ± 0.22	0.960 ± 0.27
6.0 mm horizontal CVI	63.94 ± 4.0%	63.89 ± 3.7%	64.36 ± 3.5%	63.55 ± 4.3%	64.64 ± 3.3%	62.88 ± 4.1%	65.05 ± 3.4%
6.0 mm horizontal LA	0.947 ± 0.37	1.057 ± 0.37	1.045 ± 0.40	1.003 ± 0.35	1.064 ± 0.33	0.905 ± 0.27	1.140 ± 0.30
6.0 mm horizontal SA	0.532 ± 0.21	0.596 ± 0.23	0.572 ± 0.22	0.566 ± 0.19	0.579 ± 0.18	0.535 ± 0.17	0.607 ± 0.15
6.0 mm horizontal TCA	1.478 ± 0.56	1.654 ± 0.59	1.617 ± 0.61	1.569 ± 0.52	1.643 ± 0.49	1.440 ± 0.42	1.747 ± 0.43
6.0 mm vertical CVI	63.56 ± 4.0%	63.58 ± 3.6%	64.29 ± 3.7%	63.79 ± 4.5%	64.12 ± 3.8%	64.59 ± 3.6%	66.36 ± 3.6%
6.0 mm vertical LA	1.103 ± 0.41	1.263 ± 0.43	1.239 ± 0.46	1.200 ± 0.40	1.255 ± 0.37	1.082 ± 0.31	1.365 ± 0.31
6.0 mm vertical SA	0.626 ± 0.22	0.717 ± 0.25	0.675 ± 0.24	0.662 ± 0.19	0.697 ± 0.20	0.590 ± 0.16	0.688 ± 0.16
6.0 mm vertical TCA	1.729 ± 0.62	1.980 ± 0.67	1.914 ± 0.69	1.861 ± 0.58	1.952 ± 0.55	1.672 ± 0.46	2.053 ± 0.45

CVI, choroidal vascular index; TCA, total choroidal area; LA, luminal area; SA, stromal area.

**Table 5:** Comparison of retinal and choroidal parameters at different time points.

	Preoperative vs. Post-Week 1	p *	Post-Month 1 vs. Post-Month 12	p *
Eyes (*n*)	69 (*n* = 35)		19 (*n* = 10)	
CRT (μm)	271.49 ± 31.6 vs. 284.69 ± 36.9	<0.0001	277.11 ± 22.9 vs. 284.68 ± 33.7	<0.0001
3 mm CRV (μm^2^)	0.213 ± 0.02 vs. 0.224 ± 0.03	<0.0001	0.218 ± 0.02 vs. 0.223 ± 0.03	<0.0001
6 mm CRV (μm^2^)	8.817 ± 0.67 vs. 8.900 ± 1.04	<0.0001	8.75 ± 0.35 vs. 8.97 ± 0.55	<0.0001
Subfoveal ChT	195.13 ± 88.4 vs. 184.86 ± 84.2	<0.0001	193.68 ± 59.1 vs. 202.58 ± 64.0	<0.0001
0.5 mm nasal ChT	190.77 ± 92.3 vs. 178.41 ± 89.7	<0.0001	189.11 ± 62.8 vs. 195.47 ± 66.6	<0.0001
1.5 mm nasal ChT	162.30 ± 78.3 vs. 148.90 ± 77.4	<0.0001	151.95 ± 65.7 vs. 158.11 ± 52.3	<0.0001
3.0 mm nasal ChT	121.05 ± 50.7 vs. 102.02 ± 55.3	<0.0001	102.13 ± 48.6 vs. 106.06 ± 52.3	<0.0001
0.5 mm temporal ChT	211.31 ± 95.8 vs. 203.97 ± 86.4	<0.0001	205.32 ± 59.0 vs. 216.00 ± 58.9	<0.0001
1.5 mm temporal ChT	280.99 ± 95.4 vs. 265.71 ± 86.3	<0.0001	270.89 ± 60.4 vs. 279.16 ± 67.0	<0.0001
3.0 mm temporal ChT	355.37 ± 92.3 vs. 318.50 ± 77.9	<0.0001	332.68 ± 74.4 vs. 333.00 ± 84.6	<0.0001
0.5 mm Superior ChT	240.72 ± 98.8 vs. 214.62 ± 92.7	<0.0001	220.63 ± 58.0 vs. 228.89 ± 60.1	<0.0001
1.5 mm Superior ChT	287.04 ± 88.7 vs. 262.48 ± 88.7	<0.0001	256.95 ± 50.7 vs. 264.74 ± 54.9	<0.0001
3.0 mm Superior ChT	350.22 ± 104.3 vs. 316.36 ± 100.0	<0.0001	323.89 ± 85.2 vs. 332.47 ± 73.9	<0.0001
0.5 mm inferior ChT	204.44 ± 90.0 vs. 190.68 ± 90.1	<0.0001	196.32 ± 63.4 vs. 203.84 ± 64.8	<0.0001
1.5 mm inferior ChT	220.58 ± 91.3 vs. 217.65 ± 92.8	<0.0001	218.21 ± 71.8 vs. 229.74 ± 82.0	<0.0001
3.0 mm inferior ChT	271.94 ± 114.8 vs. 263.58 ± 95.2	<0.0001	285.65 ± 99.8 vs. 299.41 ± 96.0	<0.0001
3.0 mm horizontal CVI	65.42% ± 3.9% vs. 66.08% ± 4.0%	<0.0001	67.28% ± 5.0% vs. 68.07% ± 5.3%	<0.0001
3.0 mm horizontal LA	0.497 ± 0.22 vs. 0.558 ± 0.24	<0.0001	0.613 ± 0.17 vs. 0.644 ± 0.18	<0.0001
3.0 mm horizontal SA	0.262 ± 0.12 vs. 0.283 ± 0.12	<0.0001	0.297 ± 0.09 vs. 0.302 ± 0.09	<0.0001
3.0 mm horizontal TCA	0.760 ± 0.34 vs. 0.841 ± 0.35	<0.0001	0.910 ± 0.25 vs. 0.946 ± 0.25	<0.0001
3.0 mm vertical CVI	65.48% ± 3.7% vs. 65.90% ± 4.5%	<0.0001	67.28% ± 5.0% vs. 67.75% ± 5.0%	<0.0001
3.0 mm vertical LA	0.533 ± 0.23 vs. 0.600 ± 0.24	<0.0001	0.613 ± 0.17 vs. 0.644 ± 0.18	<0.0001
3.0 mm vertical SA	0.279 ± 0.12 vs. 0.308 ± 0.12	<0.0001	0.319 ± 0.09 vs. 0.323 ± 0.11	<0.0001
3.0 mm vertical TCA	0.812 ± 0.34 vs. 0.908 ± 0.35	<0.0001	0.977 ± 0.25 vs. 1.002 ± 0.28	<0.0001
6.0 mm horizontal CVI	64.62% ± 4.0% vs. 64.36% ± 3.5%	<0.0001	67.28% ± 5.0% vs. 67.75% ± 5.0%	<0.0001
6.0 mm horizontal LA	0.930 ± 0.40 vs. 1.045 ± 0.40	<0.0001	0.613 ± 0.17 vs. 0.644 ± 0.18	<0.0001
6.0 mm horizontal SA	0.507 ± 0.22 vs. 0.572 ± 0.22	<0.0001	0.297 ± 0.09 vs. 0.302 ± 0.09	<0.0001
6.0 mm horizontal TCA	1.437 ± 0.62 vs. 1.617 ± 0.61	<0.0001	0.910 ± 0.25 vs. 0.946 ± 0.25	<0.0001
6.0 mm vertical CVI	63.96% ± 4.2% vs. 64.29% ± 3.7%	<0.0001	67.28% ± 5.0% vs. 68.07% ± 5.3%	<0.0001
6.0 mm vertical LA	1.087 ± 0.45 vs. 1.239 ± 0.46	<0.0001	0.658 ± 0.17 vs. 0.679 ± 0.18	<0.0001
6.0 mm vertical SA	0.606 ± 0.24 vs. 0.675 ± 0.24	<0.0001	0.319 ± 0.09 vs. 0.323 ± 0.11	<0.0001
6.0 mm vertical TCA	1.693 ± 0.68 vs. 1.914 ± 0.69	<0.0001	0.977 ± 0.25 vs. 1.002 ± 0.28	<0.0001

* Determined by a repeated measures mixed-effects model, with *p* < 0.05 considered statistically significant.

## Data Availability

The data supporting the results of the current study can be found within the article.
